# Health information anxiety in social media users during public health emergencies: A qualitative comparative analysis using attribution theory

**DOI:** 10.1371/journal.pone.0340674

**Published:** 2026-01-29

**Authors:** Xiao Wenchang, Yang Xuanhui, Zeng Qun, Cheng Xiao

**Affiliations:** School of Public Policy and Administration, Nanchang University, Jiangxi, China; King George's Medical University, INDIA

## Abstract

This article investigates the mechanisms influencing health information anxiety among social media users during sudden public health emergencies, aiming to provide insights for managing social media users’ negative emotions in such contexts. By employing literature analysis and case studies and integrating Three-Dimensional Attribution Theory, the factors contributing to health information anxiety are classified into individual, informational, and situational dimensions. Questionnaire data were gathered via scenario simulation, and a Qualitative Comparative Analysis (QCA) method was used to validate causal configurations leading to health information anxiety among social media users. The findings indicate that, within the context of sudden public health emergencies, the emergence of health information anxiety is the result of the interplay among individual, situational, and informational dimensions. Specifically, six key factors, including event severity, involvement, textual sentiment, collective emotions, information overload, and information asymmetry, are identified as playing a critical role in the development of severe health information anxiety. Notably, the situational dimension is found to exert a crucial and decisive influence on the generation of health information anxiety among social media users.

## 1. Introduction

A sudden public health emergency refers to an event that “seriously affects public health, such as the sudden outbreak of a major infectious disease, unexplained mass diseases, major food or occupational poisoning, etc.” [1]. The exponential increase of social media information makes it difficult for users to distinguish and digest the inundation of health information on disease prevention, wellness, healthcare services, etc., and the resulting feelings of unease and tension are called health information anxiety [2]. The dissemination of social media information is complex and unpredictable, and its convenient interactive nature provides a crucial channel for public demands and emotional expression during sudden events. Public emotional expressions on social media not only facilitate the spread of public opinion information but also accelerate emotional contagion among the public, leading to the rapid spread of collective emotions on social media.

In the context of sudden public health emergencies, the improper dissemination of online health information not only adversely affects individual health conditions but also poses a risk of inducing societal public health panic. Health information anxiety, as a negative emotion generated by social media users in the context of sudden public health emergencies, forms the basis for the emergence of online public sentiment. In occurrences of sudden public health emergencies, with the dissemination of information, the emotions of netizens propagate rapidly on the internet, accelerating the formation of online group polarization phenomena. This process leads to the emergence of health information anxiety among social media users and even amplifies the panic emotions of netizens. Consequently, it results in the uncontrollable escalation of online public opinion and impacts societal stability.

Therefore, this study employs the Qualitative Comparative Analysis method to investigate the underlying mechanisms influencing health information anxiety among social media users in the context of sudden public health emergencies. The results provide insights that may serve as guidelines for mitigating negative emotions among social media users during outbreaks caused by sudden public health crises.

## 2. Literature review and research framework

### 2.1. Literature review

The collective influence of various factors during the online health information search process has led to the emergence of health information anxiety among users [3]. Moreover, users’ levels of health information awareness and health anxiety significantly influence their online health information-seeking behavior. Concurrently, such information-seeking behavior may reciprocally intensify the user’s health information anxiety [4]. The disparity in the public’s comprehension of health information and the negative perceptions surrounding the generation of health information constitute critical factors contributing to the increase in public health information anxiety [5]. Health information anxiety further influences users’ risk perception, emotional responses, and behaviors [6]. Meanwhile, factors such as information overload, information conflict, information narrowing, and information filter bubbles on social media can induce health information anxiety during the health information search process [7,8]. When users are exposed to health information beyond their processing capacity, their perception of health risks and negative emotions regarding their health often intensify, subsequently leading to avoidance behaviors concerning health information [9]. The level of users’ health information anxiety is significantly associated with the duration and frequency of their online health information-seeking behavior [10]. Additionally, factors such as the quality of health information and the credibility of information sources can influence users’ perceptions of information, further contributing to health-related anxiety [11].

Although existing research has extensively explored the factors contributing to users’ health information anxiety from various perspectives, most studies have predominantly focused on examining the relationships between individual influencing factors. There remains a significant gap in understanding the mechanisms underlying health information anxiety, particularly regarding the configurational relationships arising from interactions between multiple factors. Therefore, this study employs the Qualitative Comparative Analysis method to investigate the influencing factors and their configurational relationships contributing to health information anxiety among social media users during sudden public health emergencies. This study aims to identify the antecedent conditions and causal configurations that contribute to health information anxiety, thereby uncovering the mechanisms behind its emergence during public health emergencies.

### 2.2. Theoretical background and analytical framework

Three-Dimensional Attribution Theory, introduced by Kelly in 1967 as a psychological framework for investigating individual behavior, posits that the causes of individual behavior can be analyzed through three dimensions: the actor, the objective stimulus leading to the behavior, and the background or context in which the behavior occurs [12]. When exploring the factors influencing the behavior of information subjects in the context of this study, the actor in Three-Dimensional Attribution Theory can be understood as the individual dimension, the objective stimulus as the informational dimensions, and the background or context of behavior as the situational dimensions. Thus, an individual’s information behavior can be regarded as the result of the interactive influence of these three elements. In relation to Three-Dimensional Attribution Theory, researchers have applied it to scenarios of sexual harassment. This involves determining how observers utilize consistency, distinctiveness, and consensus information to ascertain whether the target or perpetrator should be held accountable for instances of sexual harassment [13]. Additionally, research has expanded the application of Three-Dimensional Attribution Theory into the Role Congruity Theory, providing a more comprehensive framework to explain how the gender of leaders moderates the relationship between leadership styles and subordinate performance [14]. Furthermore, studies have integrated this theory with the Information Systems Success Model to construct a novel theoretical framework. This framework has been tested to assess users’ satisfaction with the system experience [15]. This paper, grounded in Three-Dimensional Attribution Theory, integrates the characteristics of individual factors, informational factors, and situational factors. It analyzes the antecedent conditions and configurations leading to the generation of health information anxiety among social media users during sudden public health emergencies.

#### 2.2.1. Theoretical framework based on Three-Dimensional Attribution Theory.

**Fig 1:**
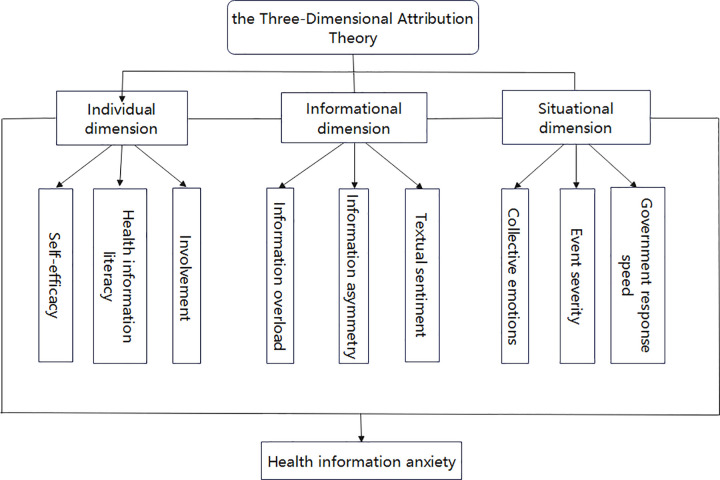
Analysis framework of factors influencing health information anxiety among social media users during public health emergencies.

The generation of health information anxiety is intricately linked to individual, situational, and informational dimensions. Grounded in Three-Dimensional Attribution Theory, this paper categorizes the influencing factors of health information anxiety among social media users during sudden public health emergencies into three dimensions: individual, situational, and informational. Drawing upon case analyses and a review of existing literature, the paper introduces nine variables: self-efficacy, health information literacy, involvement, information asymmetry, information overload, textual sentiment, collective emotions, event severity, and government response speed. These variables form the basis for constructing a three-dimensional analytical framework comprising nine indicators for the analysis of factors influencing health information anxiety among social media users during sudden public health emergencies, as illustrated in Fig 1.

#### 2.2.2. Individual dimension and health information anxiety.

In the impact factor framework model constructed in this paper, the individual dimension encompasses three aspects: self-efficacy, health information literacy, and involvement.

Bandura proposes that self-efficacy refers to an individual’s ability to engage in a specific activity within a particular context and achieve the expected outcomes; self-efficacy largely reflects the individual’s perception of their own capabilities [16]. Individuals with high self-efficacy are more capable of recovering rapidly from setbacks, and they can further improve their cognitive processes and attitudes towards things, ultimately achieving their goals [17]. During sudden public health emergencies, health information related to these incidents on social media may trigger excessive attention or heightened sensitivity among users. Due to a lack of knowledge about relevant health information, when users perceive themselves as incapable of coping with the risks, it can easily lead to health information anxiety among social media users.

Health information literacy refers to an individual’s capacity to collect, discern, and utilize health information through various channels [18]. Health information literacy can impact the health decision-making behavior of social media users. During sudden public health emergencies, social media users are surrounded by a plethora of health information that is difficult to distinguish, may be conflicting, and overwhelms them with an information overload, making it more challenging for users to assess the authenticity of health information. Insufficient health information literacy hinders the public’s ability to discern the authenticity of health information during sudden public health emergencies, which may contribute to collective panic. Users with higher health information literacy can discern valuable and genuine health information from the complex health information environment and take effective measures, thereby reducing their health information anxiety.

The concept of involvement, initially introduced by Sherif et al., signifies an individual’s perception of the degree to which an event is relevant to oneself [19]. Palanisamy et al. extended the concept of involvement into the consumer behavior domain, defining it as the consumer’s perception of the degree of association between external stimuli and their personal needs [20]. In the context of this paper, involvement is defined as the individual’s perception of the relevance and importance of external matters to them when facing external environmental stimuli during sudden public health emergencies. Previous research indicates that involvement plays a crucial role in the generation and dissemination of rumors [21].

#### 2.2.3. Informational dimension and health information anxiety.

The informational dimension in the theoretical model constructed in this paper primarily consists of information overload, information asymmetry, and textual sentiment.

Information overload refers to a state in which the amount of information encountered by the public far exceeds the individual’s capacity for processing, transforming, and assimilating, to the extent that the audience is unable to accurately receive, process, transform, and apply effective information [22]. Information overload emphasizes the psychological and emotional imbalance that users experience when faced with a plethora of information online due to insufficient processing capabilities. This imbalance can impact users’ objective judgment, rational cognition, and even influence their health information behavior and decision-making [23]. During sudden public health emergencies, social media serves as a crucial channel for people to acquire information, engage in discussions, and express emotions. The platform is inundated with information of uncertain origin, making it challenging for individuals to verify the objectivity and authenticity of the information. This leads to users experiencing difficulty in making choices and experiencing emotional stress, contributing to feelings of unease and anxiety during the process of information processing on social media.

Existing research indicates that information asymmetry is a primary factor leading to the development of public social emotions during the evolution of public opinion and serves as a direct factor causing changes in public sentiment. In the context of sudden public health emergencies, information asymmetry contributes to the emergence of anxiety among social media users, while such anxiety subsequently amplifies public risk perception regarding the event [21]. The forms of expression in health information dissemination, such as fear appeals, can trigger emotional responses among social media users, consequently leading to the generation of health information anxiety [24]. In this paper, textual sentiment refers to the emotion conveyed to users in health information texts on social media. The textual sentiment of health information during sudden public health emergencies is classified into two categories: positive and negative sentiment.

#### 2.2.4. Situational dimension and health information anxiety.

In the constructed framework model of influencing factors in this paper, the situational dimension comprises three elements: collective emotions, event severity, and government response speed.

Collective emotion in this paper refers to the emotions expressed by the public when voicing opinions on social media. Emotions, as a form of specialized information, generally exhibit contagious characteristics. In the context of sudden emergencies, the information prevalent on social media often tends to feature weak information and strong emotions. During sudden public health emergencies, public judgments are often influenced by collective emotions, where heightened collective emotions may lead to the occurrence of excessive behavior among social media users [25]. Netizens are highly susceptible to the influence of online public opinion during emergencies, resulting in feelings of unease and tension. These negative emotions, spread through the dissemination of public opinion messages, trigger emotional resonance among more social media users, leading to the outbreak and spread of negative emotions on social media through the mechanism of emotional contagion. As a result, it exacerbates health information anxiety among social media users during sudden public health emergencies.

Event severity refers to the degree of health threat and the extent of impact on the public caused by a sudden public health emergency. A higher event severity indicates a greater degree of harm, a broader scope of impact, and a higher likelihood of eliciting negative emotions among the public [26].

Government response speed refers to the promptness with which the government provides a positive response following the occurrence of a sudden public health emergency. As an integral component of the external environment, the speed of government response during the outbreak of sudden public health emergencies can influence the intensity of negative emotions among the public [27].

## 3. Methodology

### 3.1. Research method

The Qualitative Comparative Analysis method differs from previous research by not solely focusing on the impact of individual factors on results. QCA posits that a particular outcome arises from the combined influence of multiple factors, and the impact of individual variables on outcomes is limited [28]. The QCA method explores multiple concurrent relationships between variables from a configurational perspective, providing an effective means to investigate complex causal relationships among factors contributing to a particular social phenomenon [29]. Qualitative Comparative Analysis is principally classified into three types: crisp-set QCA (csQCA), multi-value QCA (mvQCA), and fuzzy-set QCA (fsQCA). csQCA is typically employed for handling dichotomous variables, whereas mvQCA is appropriate for analyzing multi-categorical variables. In contrast, fsQCA is well-suited for analyzing continuous variables. In particular, fsQCA’s consistency evaluation mechanism enhances the precision of set-theoretic analysis. Based on the analysis of survey data, this study explores the underlying mechanisms of public health information anxiety in the context of public health emergencies. Compared with csQCA and mvQCA, the fsQCA approach offers a better methodological fit for this research.

### 3.2. Questionnaire survey

This study investigates the impact of various variables on health information anxiety through questionnaire data collection. A scenario-based simulation approach was employed to evoke subjects’ memories, using materials adapted from emergent pandemic events. The initial section of the questionnaire consisted of an informed consent statement, outlining the purpose and significance of the study, emphasizing the anonymity and voluntariness of participation. All information provided by respondents was treated with strict confidentiality and used exclusively for academic research purposes. Participants were required to carefully review the provided materials and respond to questionnaire items based on their authentic perceptions. Ethical approval for this survey study was deemed unnecessary by Nanchang University, as the research involved minimal risk and complied with the institutional ethical standards for social science research.

The questionnaire items in this study were adapted from established items in prior studies, and each variable item utilized a 7-point Likert scale. For the survey conducted in China, the research team—comprising members fluent in Chinese and English—carefully translated the questionnaire into Chinese [30]. After finalizing the design of the measurement scales, the survey was implemented online through the Wen-juan-wang survey platform. In order to ensure validity, the research team conducted a pilot survey with 60 participants, followed by a reliability and validity analysis of the collected data. The design and wording of several questionnaire items were subsequently revised based on the analytical results and respondent feedback. The variables and corresponding questionnaire items are outlined in Table 1.

**Table 1:** Measuring items for research model.

Constructs	Items	Reference Source
Health information anxiety (HIA)	HIA1: I experience a sense of unease when confronted with unforeseen public health crises.	[31,32]
	HIA2: I experience a heightened level of tension when faced with sudden public health emergencies.	
	HIA3: I feel a sense of relaxation when reviewing information related to unexpected public health emergencies (reverse-coded).	
Self-efficacy (SE)	SE1: I am adept at self-protection when faced with unexpected public health emergencies.	[33]
	SE2:Mitigating the impact of unforeseen public health emergencies on myself is not a challenging task.	
	SE3: I am knowledgeable about taking effective measures to prevent the impact of unforeseen public health emergencies on myself.	
Health information literacy (HIL)	HIL1: I can accurately distinguish between credible and non-credible health information when exposed to multiple health-related messages.	[34,35]
	HIL2: I demonstrate acute information awareness by maintaining skepticism toward unverified online health claims.	
	HIL3: I can efficiently locate, comprehend, and apply relevant health information from available sources.	
Involvement (IV)	IV1: Sudden public health emergencies are relevant to me.	[36]
	IV2: Sudden public health emergencies are pertinent to me.	
	IV3: Sudden public health emergencies are not significantly relevant to me (reverse-coded).	
Information overload (IO)	IO1: There is an abundance of information regarding sudden public health emergencies on the internet, and comprehending all of it is challenging for me.	[37]
	IO2: The majority of information on the internet about sudden public health emergencies is not relevant to me.	
	IO3: The internet contains an unmanageable volume of information regarding sudden public health emergencies, which frequently exceeds individuals’ cognitive processing capacity.	
Information asymmetry (IA)	IA1: I perceive official media or involved entities to be withholding certain information when releasing details about sudden public health emergencies.	[38,39]
	IA2: I believe that official/involving entities have not accurately communicated the true situation of the sudden public health emergencies to the public.	
	IA 3: The information related to sudden public health emergencies held by official media/involved entities differs from what I possess.	
Textual sentiment (TS)	TS1: The wording used in online information about sudden public health emergencies is consistently relaxing to read. (reverse-coded).	[40]
	TS2: The language of sudden public health emergencies information online is typically anxiety-provoking.	
	TS3: Online information about emergent public health emergencies consistently uses distressing terminology.	
Event severity (ES)	ES1: I perceive this sudden public health emergency as severe.	[41]
	ES2: I believe that this particular sudden public health emergency will lead to severe consequences.	
	ES3: I do not perceive this particular sudden public health emergency as critical (reverse-coded).	
Collective emotions (CE)	CE1: The information publicly shared online makes me perceive that the public is uneasy.	[42]
	CE2: The information publicly shared online makes me perceive that the public is anxious.	
	CE3: The information publicly shared online about sudden public health emergencies makes me feel that their emotions are generally positive (reverse-coded).	
Government response speed (GRS)	GRS1: After the outbreak of a sudden public health emergencies, I believe the government’s response speed is quite rapid.	[43]
	GRS2: I consider the government’s response to be timely following the emergence of a public health crisis.	
	GRS3: I consider the government’s emergency response to be untimely following the onset of a public health incident(reverse-coded).	

### 3.3. Data collection

The formal data collection process commenced on November 20, 2023, and concluded on January 6, 2024, spanning a period of approximately six weeks. Data collection was carried out using a mixed online–offline strategy. The offline stage adopted a “random interception–oral inquiry–gift provision” procedure at several universities around Nanchang, where the field survey was implemented. In this stage, undergraduate students were randomly approached, resulting in the distribution of 232 questionnaires, of which 196 were successfully retrieved. Two weeks after the commencement of the offline survey, an online snowball sampling method was introduced via https://www.wenjuan.com/ to recruit respondents already engaged in the workforce. Participation in the survey was entirely voluntary, and respondents were informed in advance that careful completion of the questionnaire would allow them to enter a lottery for cash rewards. To safeguard data quality, attention-check items were incorporated into the instrument. Subsequent post-hoc analyses confirmed no significant differences between online and offline responses with respect to measurement items. In total, 411 questionnaires were gathered (215 online and 196 offline). After eliminating invalid cases, such as those with less than one year of platform usage, identical answers across all items, failure on attention-check items, or completion times shorter than 180 seconds, 273 valid questionnaires remained for analysis, meeting the minimum case requirements for nine conditional variables. Demographic characteristics about the sample are presented in Table 2.

**Table 2:** Demographic characteristics.

Category	Sub Category	Valid responses (n = 273)		Invalid responses (n = 138)	
		Frequency	Percent (%)	Frequency	Percent (%)
Gender	Male	103	37.73	92	66.67
	Female	170	62.27	46	33.33
Age (years)	<18	1	0.37	0	0
	18 ~ 30	238	87.18	121	87.68
	31 ~ 40	26	9.52	14	10.14
	41 ~ 50	2	0.73	2	1.45
	>50	6	2.20	1	0.72
Education	Below bachelor degree	11	4.03	9	6.52
	Bachelor’s degree	137	50.18	93	67.39
	Master degree or above	125	45.79	36	26.09

### 3.4. Reliability and validity test

The reliability of the scale is generally assessed by the magnitude of Cronbach’s Alpha, and when Cronbach’s Alpha coefficient exceeds 0.7, the scale is considered to have good reliability [44]. In this study, SPSS 26 was utilized to conduct a reliability and validity test on the scale. Cronbach’s Alpha was employed to measure the reliability of the questionnaire, while the Kaiser-Meyer-Olkin (KMO) value and the significance level of Bartlett’s sphericity test were used to assess the validity of the questionnaire, as detailed in Table 3. The minimum value of Cronbach’s Alpha in the table is 0.727, indicating good reliability of the questionnaire. To examine the structural validity of the scale, an exploratory factor analysis was performed on the questionnaire data, yielding a KMO value of 0.775, exceeding 0.7, and a Bartlett sphericity test significance level of 0.000, indicating suitability for factor analysis. Therefore, the questionnaire scale demonstrates good structural validity.

**Table 3:** Reliability and validity indices.

Constructs	Cronbach’s Alpha	KMO	Bartlett Sphere Test Sig.
Health information anxiety	0.839	0.775	0.000
Self-efficacy	0.811		
Health information literacy	0.830		
Involvement	0.936		
Information overload	0.727		
Information asymmetry	0.863		
Textual sentiment	0.855		
Event severity	0.908		
Collective emotions	0.872		
Government response speed	0.919		

### 3.5. Variable explanation and data calibration

Before conducting the data analysis using the fsQCA method, it is necessary to calibrate the data. In this study, the three-valued fuzzy set calibration method was employed, involving full membership, full non-membership, and the crossover point, transforming variable values into a range between 0 and 1. For the calibration of data in this research, the scores of various items measuring the same variable were averaged. The maximum, minimum, and average values of these averages were then selected as “full membership,” “full non-membership,” and “crossover point,” respectively, as illustrated in Table 4.

**Table 4:** Data calibration table.

Constructs	Set of objectives	Full membership	Crossover point	Full non-membership
Health information anxiety	severe health information anxiety	7	4.6899	1
Self-efficacy	high self-efficacy	7	5.9158	3.33
Health information literacy	high health information literacy	7	5.2186	3
Involvement	high involvement level	7	5.4103	1
Information overload	severe information overload	6.67	3.6716	1
Information asymmetry	severe information asymmetry	7	3.4005	1
Textual sentiment	intense negative emotional tone in the text	7	4.0997	1
Event severity	high event severity	7	5.2112	1
Collective emotions	intense collective negative emotions	7	4.3773	1
Government response speed	rapid governmental response	7	5.7485	1

## 4. Data analysis

### 4.1. Necessary condition testing

After the completion of data calibration, an analysis of the necessity of individual conditional variables is required before proceeding to the analysis of the truth table. This analysis is primarily based on the levels of consistency and coverage [45]. When the consistency value exceeds 0.8, the conditional variable is considered a sufficient condition for the occurrence of the dependent variable; when the consistency value exceeds 0.9, the conditional variable can be deemed a necessary condition for the occurrence of the dependent variable [28]. The coverage level indicates the degree to which a particular conditional variable or a set of conditional variables explains the outcome variable. If the coverage indicator approaches 1, it indicates that the explanatory variable can explain the dependent variable better.

This study utilized fsQCA 3.0 software for necessity testing and truth table analysis. The results of the necessity test are presented in Table 5, where “~” signifies the absence of a particular condition. From the results in the table, it can be observed that the consistency values of involvement, event severity, and government response speed all exceeded 0.8. High involvement, significant event severity, and rapid government response speed are sufficient conditions leading to severe health information anxiety among social media users during sudden public health emergencies. There are no single variables identified as necessary conditions for severe health information anxiety.

**Table 5:** Single variable necessity test.

Conditional variables	Outcome variable (Health Information Anxiety)				
	HIA			~HIA	
	Consistency		Coverage	Consistency	Coverage
Self-efficacy	SE	0.7690	0.7165	0.7652	0.5998
	~SE	0.5705	0.7428	0.6383	0.6992
Health information literacy	HIL	0.7030	0.7356	0.7272	0.6402
	~HIL	0.6561	0.7408	0.6996	0.6647
Involvement	IV	**0.8181**	0.7322	0.7694	0.5794
	~IV	0.5300	0.7320	0.6444	0.7488
Information overload	IO	0.7105	0.7783	0.7325	0.6751
	~IO	0.7034	0.7576	0.7594	0.6882
Information asymmetry	IA	0.6597	0.7586	0.7027	0.6799
	~IA	0.7216	0.7426	0.7505	0.6498
Textual sentiment	TS	0.7682	**0.8254**	0.7117	0.6434
	~TS	0.6681	0.7337	**0.8069**	0.7455
Event severity	ES	**0.8234**	0.7794	0.7315	0.5826
	~ES	0.5590	0.7122	0.7230	0.7750
Collective emotions	CE	0.7858	**0.8232**	0.7084	0.6243
	~CE	0.6414	0.7233	0.7994	0.7585
Government response speed	GRS	**0.8103**	0.7126	**0.8240**	0.6097
	~GRS	0.5563	0.7897	0.6117	0.7306

In situations where public health information anxiety does not occur, the consistency values for ~textual sentiment and government response speed are 0.807 and 0.824, respectively. This indicates that positive textual sentiment and rapid government response speed are sufficient conditions for social media users to avoid health information anxiety during sudden public health emergencies. Similarly, no single conditional variable is identified as a necessary condition for social media users to avoid health information anxiety during sudden public health emergencies. This implies the absence of individual conditions causing user health information anxiety, highlighting the necessity for subsequent configurational analysis to extract the combinations of conditional variables leading to health information anxiety among social media users during sudden public health emergencies.

### 4.2. Conditional combination analysis

This study employed fsQCA 3.0 software to construct a truth table for analyzing the configurations leading to health information anxiety among social media users during sudden public health emergencies. During the truth table analysis, the case frequency threshold is typically set to 1. When the sample size is substantial, it may be necessary to raise the case threshold. Ragin suggests that when setting the case threshold, it should be ensured that the final sample size used for analysis comprises over 90% of the total sample size [28]. In this study, the case threshold was set to 4. The consistency threshold was set to the default value of 0.8. Ultimately, three types of solutions were obtained: complex solutions, parsimonious solutions, and intermediate solutions.

Specifically, the complex solution includes only those configurations for which empirical cases are observed and excludes all logical remainders; the parsimonious solution incorporates all logical remainders; and the intermediate solution consists of configurations with empirical cases as well as theoretically plausible logical remainders. Therefore, the intermediate solution is considered the superior solution and is typically adopted as the basis for reporting results [28]. In addition, by further examining the nested relationships between the intermediate and parsimonious solutions, one can identify the core and peripheral conditions within each configuration pathway. Core conditions refer to those that exert a decisive influence on the outcome, whereas peripheral conditions play a supporting role. In QCA, when a particular condition appears in both the parsimonious and intermediate solutions, it is regarded as a core condition; when it appears only in the intermediate solution, it is classified as a peripheral condition. Accordingly, this study employed a combined analysis of the intermediate and parsimonious solutions to identify the configurations leading to health information anxiety among social media users. Consequently, six configurations were identified as leading to severe health information anxiety among social media users during sudden public health emergencies. The results of the configurations are presented in Table 6.

**Table 6:** Analysis of configurations leading to significant health information anxiety among social media users during public health emergencies.

Constructs		Configurations					
		1	2	3	4	5	6
Individual dimension	Self-efficacy	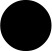	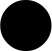	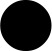	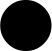	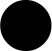	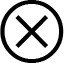
	Health information literacy	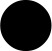	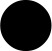		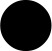	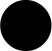	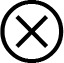
	Involvement	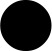	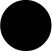	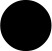	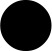	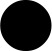	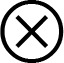
Information dimension	Information overload		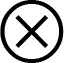	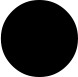	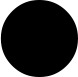	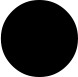	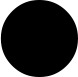
	Information asymmetry	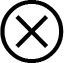		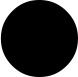	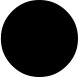	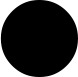	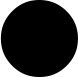
	Textual sentiment	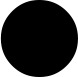	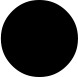	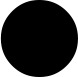	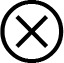	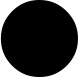	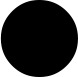
Situational dimension	Event severity	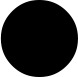	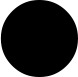	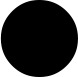	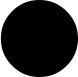	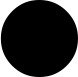	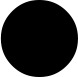
	Collective emotions	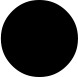	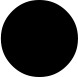	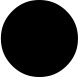	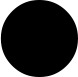	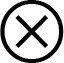	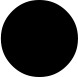
	Government response speed	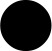	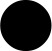	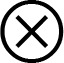	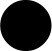	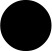	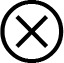
raw coverage		0.3749	0.3677	0.3120	0.3117	0.2932	0.2728
unique coverage		0.0161	0.0144	0.0263	0.0159	0.0097	0.0499
consistency		0.9465	0.9528	0.9674	0.9706	0.9764	0.9738
solution coverage		0.5343					
solution consistency		0.9363					

Note: (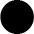 represents the occurrence of a certain condition, (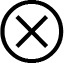 represents the absence of a specific condition. Large circles denote core conditions, small circles represent peripheral conditions, and blanks signify conditions that have no impact.

From the results in Table 6, it can be observed that the original coverage of all the configurations exceeds the unique coverage, indicating the existence of supporting cases that align with multiple causal configurations, and the coverage distribution of the configurations is relatively even. The overall consistency is 0.936, and the overall coverage is 0.534, indicating that these six configurations can explain 53.4% of the reasons for the generation of health information anxiety among social media users during sudden public health emergencies.

By observing the six configurations mentioned above, it becomes evident that high event severity appears in every configuration, indicating that the degree of health information anxiety among social media users during sudden public health emergencies is significantly influenced by the severity and scope of the event. Event severity emerges as a core condition leading to health information anxiety among social media users during sudden public health emergencies. Furthermore, the combination of self-efficacy * involvement * textual sentiment * collective emotions concurrently appears in the first three configurations. This suggests that when the event severity is high, the impact is widespread, is highly relevant to the individual, and is characterized by negative language in the textual information, the dissemination of negative emotions on social media by the public can lead to severe health information anxiety, even if social media users possess a high level of self-efficacy to mitigate the impact of the public health emergency. Additionally, information overload and information asymmetry both emerge as core conditions in the subsequent four configurations. This indicates that when the event severity is pronounced, and there is a simultaneous presence of severe information overload and information asymmetry, it is likely to result in the generation of health information anxiety among social media users.

The six configurations above further demonstrate the absence of a singular condition leading to the emergence of health information anxiety among social media users. The severe health information anxiety experienced by social media users during sudden public health emergencies results from the combined influence of individual, informational, and situational dimensions. Event severity serves as a core condition influencing the development of health information anxiety among social media users. Additionally, factors such as information overload, information asymmetry, textual sentiment, and collective emotions frequently emerge as core conditions in the configurations leading to health information anxiety, underscoring their critical role in its formation.

By setting the outcome variable as “ ~ health information anxiety “, two configurations were derived in which health information anxiety is not generated for social media users during sudden public health emergencies, in which health information anxiety is not generated. These configurations are presented in Table 7. From the table, it is evident that the overall consistency of the configurations leading to the absence of health information anxiety among social media users is 0.914, with an overall coverage rate of 0.479. This indicates that these two configurations can explain 47.9% of the reasons why social media users do not experience health information anxiety during sudden public health emergencies.

**Table 7:** Analysis of configurations for social media users not generating health information anxiety during public health emergencies.

Constructs		Configurations	
		1	2
Individual dimension	Self-efficacyv	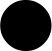	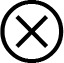
	Health information literacy	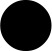	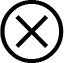
	Involvement		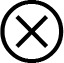
Information dimension	Information overload	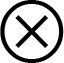	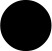
	Information asymmetry	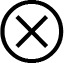	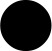
	Textual sentiment	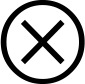	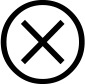
Situational dimension	Event severity	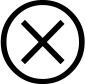	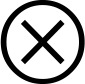
	Collective emotions	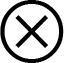	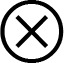
	Government response speed	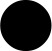	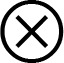
raw coverage		0.3850	0.3176
unique coverage		0.1609	0.0936
consistency		0.9245	0.9445
solution coverage		0.4785	
solution consistency		0.9137	

Observation of the above two configurations reveals that when public health emergencies demonstrate low event severity and limited spatial spread, media reports exhibit positive textual sentiment, and social media users express positive collective emotions, health information anxiety among social media users will be suppressed regardless of other factors such as the government’s timely response to the emergency, the public’s self-efficacy and health information literacy, and the presence of information overload and information asymmetry online. In other words, the combination of low event severity (~EHL), non-negative textual sentiment (~TE), and non-negative collective emotions (~GEL) itself serves as a critical condition for suppressing health information anxiety on social media during public health emergencies.

## 5. Discussion and implications

### 5.1. Discussion

This study examined the causal mechanisms of health information anxiety among social media users during sudden public health emergencies based on the Attribution Theory. A framework for the influencing factors of health information anxiety among social media users was constructed, considering individual dimension, informational dimension, and situational dimension. Data relevant to the study were collected through a scenario simulation approach. The Qualitative Comparative Analysis method was applied to analyze how the interaction of these dimensions influenced health information anxiety. The key findings are as follows:

(1) Health information anxiety during public health emergencies emerges from the conjoint influence of individual, informational, and situational dimensions. The QCA solution demonstrated a total coverage of 53.4%, with six sufficient configurations identified as contributing to health information anxiety during public health emergencies. These configurations consistently incorporated six key factors: event severity, personal involvement, textual sentiment, collective emotions, information overload, and information asymmetry. Among these factors, the event severity, textual sentiment, collective emotions, information overload, and information asymmetry simultaneously emerged consistently across both parsimonious and intermediate solutions, indicating their pivotal role for health information anxiety. The fsQCA results confirm that these factors only produce anxiety through specific combinatorial patterns, with no single condition demonstrating its sufficiency.
(2) Informational and situational dimensions play decisive roles in the generation of health information anxiety among social media users. From the configurations of health information anxiety generation among social media users, it can be observed that informational dimension, specifically information overload and information asymmetry, appear as core conditions in the final four configurations. Textual sentiment serves as a core condition in five configurations, while the situational dimension, particularly collective emotions, emerge as a core condition in five configurations. Event severity stands out as a core condition, appearing in all solution configurations. This underscores the significance of informational and situational dimensions as key determinants in the generation of health information anxiety among social media users.

Within the situational dimension, the event severity and collective emotions consistently coexist in the configurations leading to health information anxiety generation among social media users, exhibiting positive values. In contrast, these factors take on negative values in the configurations where social media users do not generate health information anxiety. This pattern suggests the crucial and determining roles of situational dimension, with event severity and collective emotions jointly fostering the generation of health information anxiety among social media users.

### 5.2. Implications

Based on the results of this study, it can be concluded that event severity is a crucial factor in the generation of health information anxiety among social media users during sudden public health emergencies. During the outbreak of public health emergencies, the aggregation, collision, and escalation of collective emotions generate a substantial protective “emotional cascade,” which accelerates the rapid dissemination of panic-driven rumors. Relevant emergency response authorities should adopt a time-critical, emotion-responsive communication strategy. Specifically, emergency management agencies must act swiftly within the golden window of crisis response to contain event escalation and mitigate emotional contagion. Measures should include rapid dissemination of accurate, authoritative information through official channels, real-time debunking of misinformation, and deployment of expert-led media briefings to stabilize public sentiment. Concurrently, emotion-sensitive content such as empathetic messaging and psychological guidance should be prioritized to alleviate public anxiety. By curbing both the objective severity of the event and the subjective emotional amplification, governments will effectively reduce the risk of widespread health information anxiety.

Simultaneously, the government should promptly alleviate the online public sentiment triggered by sudden public health emergencies, ensuring unobstructed channels and platforms for authoritative information dissemination. It is imperative to debunk panic-inducing rumors, dismantle public information barriers, prevent the emergence of negative sentiments, and steer online collective expressions toward rational and balanced directions. Authorities must vigilantly monitor evolving societal sentiments during major public emergencies. By responding to public concerns with enhanced information disclosure and actively steering public opinion, they can effectively curb rumor propagation and maintain social stability.

Finally, official media must strengthen the capacity for public opinion guidance by promptly rectifying inaccurate, ambiguous, or exaggerated reports from unverified sources. These measures would lower public risk perception during health emergencies, thereby preventing the further escalation and amplification of societal risks.

## 6. Conclusion and future prospects

This study, grounded in Attribution Theory, developed an analytical framework to investigate the factors influencing health information anxiety among social media users during sudden public health emergencies. By extracting nine explanatory variables from individual, informational, and situational dimensions, the research utilized fsQCA to explore the mechanisms underlying the emergence of health information anxiety among social media users during such crises. The study identified six configurations leading to the generation of health information anxiety and two configurations where such anxiety does not occur. The findings indicate that factors such as event severity, textual sentiment, collective emotions, information overload, and information asymmetry play pivotal roles in most configurations leading to health information anxiety. Among these factors, situational elements, specifically event severity and collective emotions, are identified as playing a crucial and decisive role in the emergence of health information anxiety. The results provide valuable insights for mitigating negative online emotions among social media users during sudden public health emergencies and offer guidance for preventing the occurrence of secondary online public opinion crises.

This study acknowledges limitations. The questionnaire-derived sample primarily consists of respondents aged 18–30, potentially incurring demographic biases. Although this age group constitutes a dominant social media cohort, such sampling bias may limit the findings’ external validity. Subsequent research should incorporate either broader population sampling or complementary methods, such as web scraping, to obtain more representative data. A further limitation of this study concerns the gender imbalance in the final sample. Although the survey was distributed through open channels with no demographic targeting, a relatively large proportion of invalid responses were identified among male participants, leading to their exclusion during data cleaning. As a result, the valid dataset contained fewer male respondents than female respondents. This imbalance may limit the generalizability of the findings across gender groups. Future research should adopt strategies to improve data quality to obtain a more gender-balanced sample. Moreover, this study employs solely the Qualitative Comparative Analysis (QCA) method to identify configurations leading to the emergence of health information anxiety, without uncovering the potential linear relationships among the variables. Future research could adopt structural equation modeling (SEM) or experimental designs to further investigate the underlying linear mechanisms among these variables. This study was conducted in the Chinese context, and individuals’ attitudes and cognitions toward public health emergencies may vary across different countries and cultures. Future research could be undertaken in other national contexts to enhance the universality of the conclusions.

## Supporting information

S1 Data Data after cleaning and calibration. (XLSX)
